# Dynamic properties of simulated brain network models and empirical resting-state data

**DOI:** 10.1162/netn_a_00070

**Published:** 2019-02-01

**Authors:** Amrit Kashyap, Shella Keilholz

**Affiliations:** Department of Biomedical Engineering, Georgia Tech and Emory University, Atlanta, GA, USA; Department of Biomedical Engineering, Georgia Tech and Emory University, Atlanta, GA, USA

**Keywords:** Resting-state fMRI, Brain network modeling, Dynamics, Dynamic functional connectivity

## Abstract

Brain network models (BNMs) have become a promising theoretical framework for simulating signals that are representative of whole-brain activity such as resting-state fMRI. However, it has been difficult to compare the complex brain activity obtained from simulations to empirical data. Previous studies have used simple metrics to characterize coordination between regions such as functional connectivity. We extend this by applying various different dynamic analysis tools that are currently used to understand empirical resting-state fMRI (rs-fMRI) to the simulated data. We show that certain properties correspond to the structural connectivity input that is shared between the models, and certain dynamic properties relate more to the mathematical description of the brain network model. We conclude that the dynamic properties that explicitly examine patterns of signal as a function of time rather than spatial coordination between different brain regions in the rs-fMRI signal seem to provide the largest contrasts between different BNMs and the unknown empirical dynamical system. Our results will be useful in constraining and developing more realistic simulations of whole-brain activity.

## Author Summary

The development of more sophisticated models of the brain will allow us to address some of the most challenging questions in neuroscience, such as how the physical structure of the brain can give rise to behavior, consciousness, and memory. Our focus in this manuscript is on simulating the relatively slow brain signals that coordinate information transfer across large scales in the brain and that can be measured using fMRI. Previous measures used averaged measures of functional connectivity in the simulated brain signals to compare with the empirical signal. In order to extend previous findings, we use dynamic analysis techniques developed for these fMRI signals to understand more transient events that occur naturally during normal brain activity. We show that these dynamic properties are better in differentiating models from each other and from the measured brain activity. These results will be useful in constraining and developing more realistic simulations of whole-brain activity.

## INTRODUCTION

The complex activity patterns produced by the brain are critical for understanding behavior and the function of the central nervous system. To explain its complexity, studies have used resting-state fMRI (rs-fMRI) scans and functional connectivity (FC) analysis to describe the coordination between different brain regions of interest (ROIs) during rest, task, and other behavioral paradigms (Smith et al., 2009). In recent years, analysis of FC data has moved beyond looking at average statistical relationships maintained over the course of a long scan (average FC) to dynamic analysis methods that assume the coordination of brain activity changes on a moment-to-moment basis (Hutchison et al., 2013; Keilholz, Caballero-Gaudes, Bandettini, Deco, & Calhoun, 2017; Shakil, Lee, & Keilholz, 2016). The anatomical connections of the brain are assumed to remain constant on these short timescales so that the time-varying coordinated activity plays out over the same framework of structural connectivity (SC) based on white matter connections over time (Cabral, Kringelbach, & Deco, 2017; Deco, Kringelbach, Jirsa, & Ritter, 2017; Shen, Hutchison, Bezgin, Everling, & McIntosh, 2015). The brain’s activity can be modeled as interactions of ROIs connected by a structural network, where the activity of eachROI is a function of the local state of processing plus the delayed activity of its network neighbors (Breakspear, 2017; Sanz-Leon, Knock, Spiegler, & Jirsa, 2015). The resulting set of differential equations form a dynamical system that can be used as a generative model to simulate activity across the whole brain for a given state vector of ROI activity. Thus, *dynamic* FC can be thought of as the brain’s trajectory across the phase space of the underlying *dynamical system* (Cabral et al., 2017; Deco et al., 2013).

Numerical simulations of this network of ROIs, known as the brain network model (BNM) (Sanz-Leon et al., 2015), simulate spovntaneous neural activity in the absence of external stimuli. Without explicit external stimuli, as in rs-fMRI, there exists no time-locked measure or event that would allow for straightforward comparison across modalities. Instead, researchers have used measures that summarize activity throughout the brain, such as average FC, estimated through Granger causality or correlation, along with distance metrics or graph theoretic analysis to quantify similarity across modalities (Cabral, Hugues, Sporns, & Deco, 2011; Liang et al., 2015; Senden, Reuter, van den Heuvel, Goebel, & Deco, 2017). At least 12 different BNMs have successfully reproduced the most prominent features of average FC (Cabral, Hugues, Kringelbach, & Deco, 2012; Cabral et al., 2011; Cabral et al., 2017; Hansen, Battaglia, Spiegler, Deco, & Jirsa, 2015; Sanz-Leon et al., 2015; Senden et al., 2017). Newer studies have tried to develop more complex BNMs to describe transient features observed in resting state, such as spontaneous switching between two FC states during rest in order to more faithfully simulate natural brain activity (Cabral et al., 2017; Deco et al., 2018; Hansen et al., 2015). However, as BNMs have become more sophisticated, dynamic analysis methods for rs-fMRI have also become more developed, leading to the question of which dynamical properties of rs-fMRI BNMs can reproduce. Many dynamic analysis methods have been applied to rs-fMRI and provide complementary views of the brain activity (Hutchison et al., 2013; Keilholz et al., 2017). The replication of these dynamic features using a generative model would provide new insight on how they might arise and what they could represent. At the same time, more stringent constraints based on dynamic rather than average rs-fMRI features would provide better discrimination between different models and between different parameterizations of the same model.

**Figure 1.**
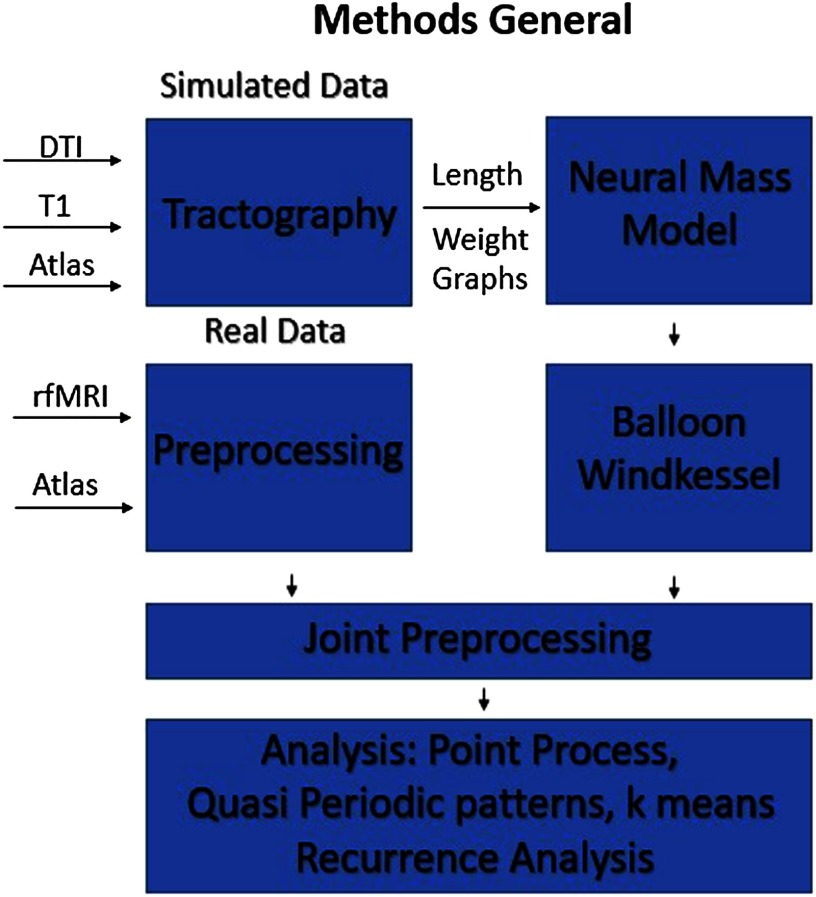
General workflow. We used DTI data to generate the length and weight matrix between ROIs of our specified atlas. Using this structural connectome, we generate data using the different neural mass models and transformed into the BOLD signal via the Balloon-Windkessel model. To compare with empirical fMRI data, scans from HCP were preprocessed and parcellated using the same atlas. The final preprocessing of filtering, global signal regression, and normalization was done jointly for all sets of data. The final data were then analyzed with each of the different dynamic analysis techniques.

The following study compares the dynamics observed in rs-fMRI to the results of the same analysis methods applied to two BNMs (Figure 1). We simulate two different types of BNMs with delayed inputs, the Kuramoto oscillator model and the Firing Rate model, and then apply four of the most common dynamic analysis techniques to compare features found in the simulated data with those found in rs-fMRI scans (Cabral et al., 2012; Cabral et al., 2011). We chose the Kuramoto and the Firing Rate models because they have been shown to be robust, have relatively few parameters to optimize, and exhibit different dynamical properties that we expect to lead to differences in analysis output (Cabral et al., 2017; Deco, Jirsa, & McIntosh, 2010). Moreover, the Firing Rate model is more simplistic in the dynamics it can reproduce, and we expect it to serve as a contrast to the more complex Kuramoto model (Cabral et al., 2017). To characterize the models and compare them with empirical data, we chose four analysis techniques that test the signal for states, repeating events, or trajectories that are representative of its higher order spatiotemporal structure. We chose these techniques in order to span the spectrum of analyzing the signal by the patterns in the spatial domain and patterns in the temporal domain. In addition, we simulated models at different parameter settings in order to understand how dynamics evolve as a function of the parameter. We used this to evaluate how well the analysis techniques compared with average FC in guiding model selection.

### Dynamic Analysis Techniques

1.  **Point process or neural avalanche theory,** which models the fMRI signal as a combination of discrete neural events or avalanches. An event in an ROI is observed when the signal crosses a threshold, and then quantifies coactivation of these events between different ROIs (Caballero et al., 2010; Liu & Duyn, 2013; Natalia, Gaudes, Dryden, Francis, & Gowland, 2012; Tagliazucchi, Balenzuela, Fraiman, & Chialvo, 2012).
2.  **Repeated or quasiperiodic spatiotemporal patterns (QPP),** which identifies a unique spatiotemporal pattern that is particularly prominent in the default mode network (DMN) and the task positive network (TPN). This pattern is extracted by iteratively using a spatiotemporal template of fixed length to correlate with the signal, finding the peaks in the correlation vector, and then averaging all the highest peaks to determine the next template (Belloy et al., 2018; Majeed et al., 2011; Majeed, Magnuson, & Keilholz, 2009; Thompson, Pan, Magnuson, Jaeger, & Keilholz, 2014; Yousefi, Shin, Schumacher, & Keilholz, 2018).
3.  **K-means clustering on windowed functional connectivity,** which identifies discrete periods in time when the spatial patterns of correlated brain activity are relatively stable. Sliding window functional connectivity matrices are clustered using k-means in order to identify the clusters in dynamic functional connectivity (Allen et al., 2014).
4.  **Recurrence quantification analysis (RQA),** which identifies repeated spatial signatures as a function of time (Webber & Marwan, 2015). In this method, the spatial pattern at each time point is correlated with the spatial pattern at all other time points, and the results are then quantified using information theory for repeated time signatures.

### Brain Network Models (Neural Mass Models)

1.  **Kuramoto model:** A model where the trajectory of each neural mass is modeled as an oscillator and the phases of each oscillator are synchronized based on network input and perturbed by random noise.
2.  **Firing Rate model:** Each neural mass is modeled by a single parameter that represents the aggregate firing rate of the population, and it decays with a certain time constant and increases its activity based on network input and random noise.

All BNMs are simulated on the same structural network but are different from each other on how they describe the evolution as a system of differential equations. Moreover, since both models have been shown to reproduce average functional connectivity, we hypothesize that the BNMs will show convergence on properties that relate more to their structural connectivity and divergence on properties that describe more their transient dynamical nature. This comparison also allows us to identify which elements of the BNMs explain particular dynamic processes observed in rs-fMRI, providing insight into the potential neurophysiological sources of types of dynamics. The results will identify metrics that enable the development of more realistic models as well as provide guidance toward the underpinnings of relevant signatures in rs-fMRI data.

## RESULTS

### Comparisons to Average Functional Connectivity

**Figure 2.**
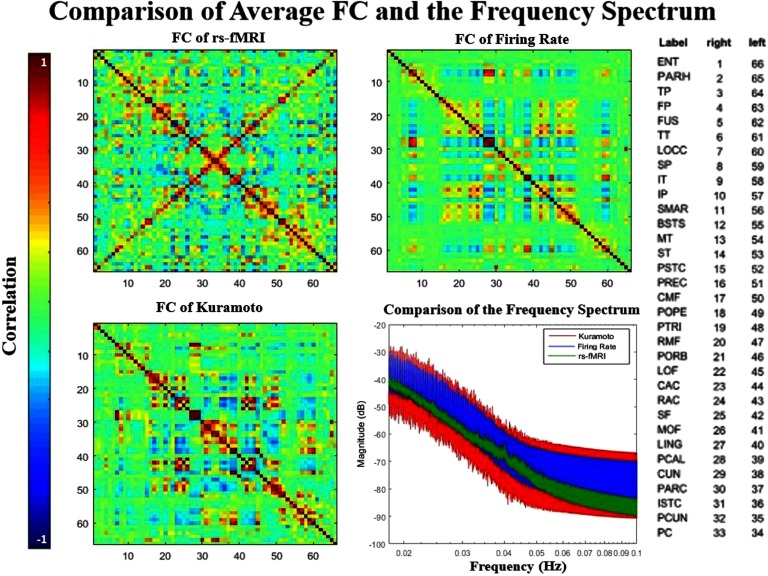
Comparison of the average functional connectivity between the rs-fMRI signals and the two simulated models. Correlation between the matrix for empirical data and the Firing Rate simulation is 0.5; correlation between empirical and Kuramoto matrices is 0.37. Both modeled matrices and the empirical data exhibit similar structure such as the coordination between hemispheres, which can be seen in the symmetry of the matrix. The mean frequency spectrum of all ROIs is plotted (bottom right) and shows that the real signal falls within range of both models. All power spectra exhibit a ${\left(\frac{1}{f}\right)}^{n}$ trend.

We first demonstrate that the simulated models reproduce common metrics in brain network modeling: average FC and power spectrum (Figure 2). The ordering of the ROIs seen in the figure is shown in Supplementary Table 1 (see Kashyap & Keilholz, 2019) and is from Cabral et al. (2011). The BNMs were simulated using the same structural connectivity as an input, randomly initialized and numerically integrated to evolve the state space according to each specific model. To approximate the BOLD signal, they are then passed through the Balloon-Windkessel model that converts neural signal to its hemodynamical response (see Methods). The methodology and parameter values are similar to those described in Cabral et al. (2011) and Cabral et al. (2012), and comparable reproduction of results are shown in Supplementary Figure 1 (Kashyap & Keilholz, 2019). We also simulated at very high and low global coupling levels to study the effect of parameterization on the other metrics. To quantify the similarity between the simulated FC matrices and the empirical FC matrix, we calculated the correlation between the two, a method that is extensively used in previous studies (Cabral et al., 2011; Cabral et al., 2017; Senden et al., 2017). Correlation was 0.37 between Kuramoto and rs-fMRI FC matrices, and 0.5 between Firing Rate and rs-fMRI FC matrices. These are in the range of values reported in earlier literature in other BNMs [0.3, 0.7] (Cabral et al., 2011; Senden et al., 2017). As expected, these correlation values are higher values than at very high and low global coupling levels (Supplementary Figure 2, Kashyap & Keilholz, 2019). Power spectra were calculated for each ROI independently, then averaged (Figure 2, bottom right). When plotted on a log-log plot, the BOLD signal has a characteristic ${\left(\frac{1}{f}\right)}^{n}$ distribution. The power exponent has been reported in literature as 0.88, comparable to the 0.9 measured here for empirical unfiltered rs-fMRI (Bullmore et al., 2000). The empirical slope falls well within the distribution of the simulated power spectrums. The two simulated models had a slope of 0.74 (Kuramoto) and 0.7 (Firing Rate) before preprocessing, comparable to a previous report of 0.78 using a different BNMs but not as good as the current best of 0.91 (Ritter, 2017; Ritter, Schirner, McIntosh, & Jirsa, 2013).

### Point Process/Neural Avalanche

**Figure 3.**
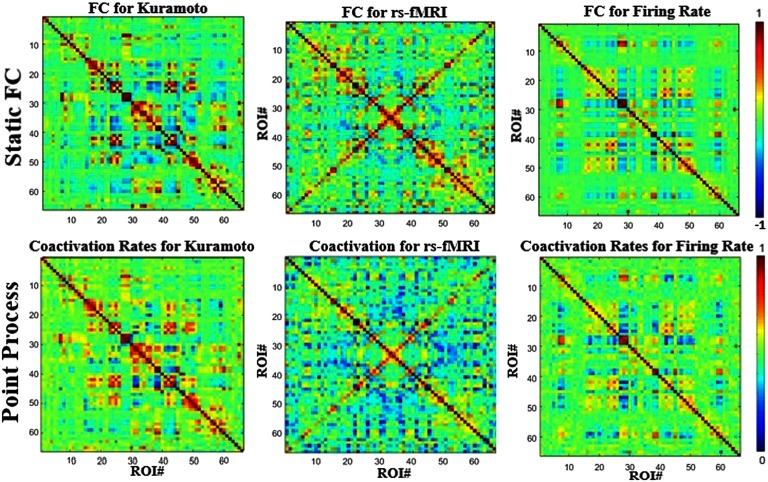
Coactivation rates (point process) between the different modalities compared with those from static or average functional connectivity. The resulting maps are almost identical between different modalities and have a correlation of over 0.9 between each respective dataset.

In coactivation analysis based on the point process approach, all ROIs that cross a certain activation threshold (see Methods; Tagliazucchi et al., 2012) are examined at each time point to identify coactivation patterns. The bottom row of Figure 3 shows the coactivation data obtained for the Kuramoto simulation, the Firing Rate simulation, and empirical rs-fMRI data. Each value in the matrix represents the fraction of co-occurrences between two ROIs. The matrices are compared with the respective average FC matrices (Figure 3, top row), and all three signals show a high degree of correlation (>0.9) between the two different analysis techniques. This is because average FC can be calculated by a handful of events, as shown by Tagliazucchi et al. (2012). Moreover, from Supplementary Figure 2 (Kashyap & Keilholz, 2019), point process/coactivation rates seem to behave identically at different parameter settings, further suggesting they are measuring similar structure in the data. The Firing Rate coactivation rates are again closer to the coactivation rates of the empirical data than the Kuramoto model, which is similar to the results observed with the average FC anaylsis.

### Quasiperiodic Pattern Algorithm Comparison

**Figure 4.**
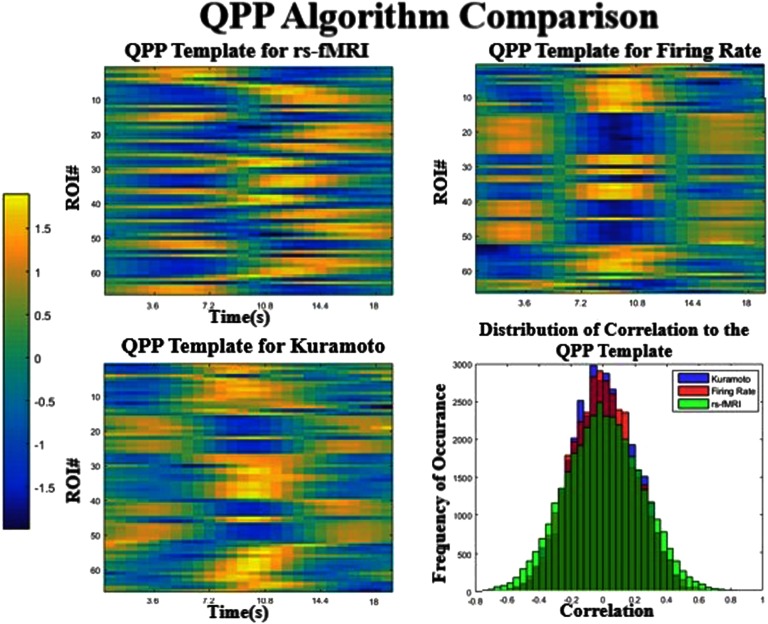
Comparison of the QPPs obtained for each model and the real data. The two simulated models (top right and bottom left) produce templates that are similar to each other but less similar to the template extracted from rs-fMRI. The correlation values to the template are plotted in a histogram (bottom right), which shows that the real signal has more extreme values than either model. All three are significantly different from each other (*p* < 0.0001) in a Komogorov-Smirnov test.

The quasiperiodic pattern (QPP) finding algorithm estimates a recurring spatiotemporal pattern that occurs throughout resting and task states. It consists of a characteristic pattern dominated by the activation and inhibition of the regions that correspond to the default mode network (DMN) and task positive network (TPN) in a specific temporal sequence (Majeed et al., 2011; Majeed et al., 2009; Yousefi et al., 2018). The QPP templates obtained from the real data and from each simulation are shown in Figure 4 in a simplified format, where the color bar shows the level of activation or deactivation in each ROI as a function of time. For better visualization, please see the supplementary videos (Kashyap & Keilholz, 2019) that show the pattern as it evolves over a surface representation of the brain. The pattern in the rs-fMRI data is consistent with the QPP templates obtained previously (Majeed et al., 2011; Yousefi et al., 2018).

The QPP templates from the two models are very similar to each other (Figure 4 top right and bottom left, correlation of 0.81), but have important differences from the empirical QPP (Figure 4, top left, correlation of 0.34, 0.33). In fact, the pattern in the simulated models seems to indicate a simple flip between two states, where a subset of ROIs is first active and then inactive. The boxy nature of the plot is due to the spatial ordering of the ROIs that was originally defined by their subnetwork connectivity, suggesting that these subcomponents are activating and deactivating together. The QPP obtained from the real data is more complex and demonstrates time lags between areas in addition to the alternation of states, suggesting that the power in the BOLD signal cyclically flows through a certain order of ROIs. The relative lengths of the simulated and the observed patterns are different as well. The QPP from the real data is approximately 20 s in length, in agreement with previous reports (Majeed et al., 2011; Yousefi et al., 2018). In contrast, both of the models give QPPs that are ∼12–13 s in length, despite the use of identical windows and the similar frequency content of the signals.

**Figure 5.**
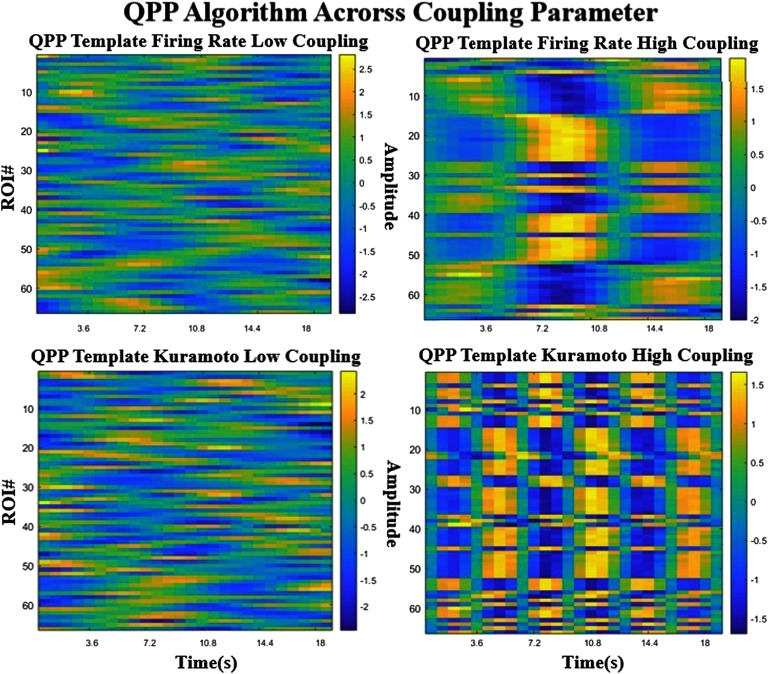
Comparison of the QPPs obtained for each model across different coupling strengths. The left column represents low global coupling and the right column, higher global coupling. The top row is the Firing Rate model and the bottom row is the Kuramoto model. As coupling strength seems to increase, the pattern goes from a random unstructured signal to a highly boxlike structure.

At low and high global coupling (Figure 5), the QPP pattern transitions from an unstructured noise-like template to a structured signal. The number of repeated patterns for a given window seems to depend on the strength of the coupling parameter for the Kuramoto model, where at higher coupling the pattern seems to shorten and repeat more often. For the Firing Model, the template pattern emerges from unstructured noise after a certain coupling strength.

The correlation vector represents how correlated the QPP template is with the scan at every time point. The distribution of the values are displayed using histograms (Figure 4, bottom right), in order to compare different modalities. The flatter distribution of the resting-state fMRI shows that the template occurs more often significantly and at higher correlations in the real scan than either of the templates.

### K-Means on Sliding Windowed Matrices

To identify FC states that occur at different time points in the BOLD signal, we used k-means analysis to compare the sliding windowed FC of the real and simulated data. After k-means clustering (k = 7), we examined both the spatial composition of the resulting clusters (or states) and metrics that describe how the brain transitions between them (Allen et al., 2014). We have also explored cluster numbers k = 8 and 9; these are shown in Supplementary Figure 3 (Kashyap & Keilholz, 2019). The results are similar across all metrics and all models. This is due to the number of actual clusters seen per individual simulation (Supplementary Figure 3, right column; Kashyap & Keilholz, 2019), which is constant across cluster numbers, suggesting that the methodology is measuring the intrinsic dynamic structure seen within the data, rather than arbitrarily dividing up the segments.

**Figure 6.**
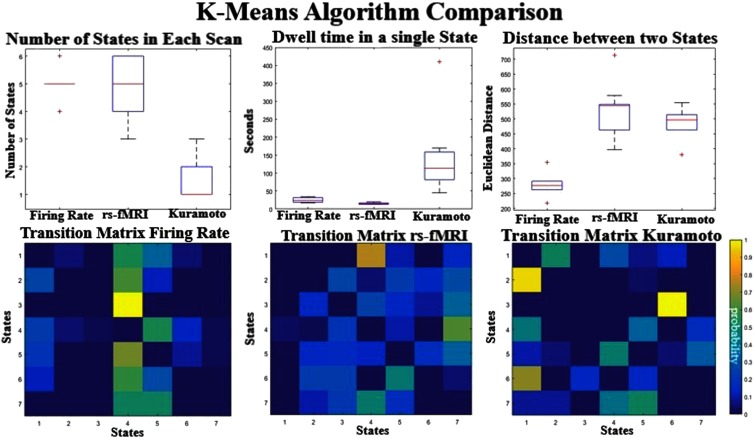
The top row shows the average number of states seen in an individual run (top left), the average dwell time in each state (top middle), and the mean distances between the centroids (top right).The transition matrices between the different k-means centroids are shown on the bottom row. The values reflect the number of raw occurrences divided by the total number of transitions giving the probability of transitioning from one state to another. Self-transitions are set to 0.

The top row in Figure 6 quantifies for each individual scan or simulation, starting at different initial conditions (*N* = 30), how many of unique states are visited, how long they dwell in each state, and how far apart (L2 Norm distance between cluster centers) these visited states are on average. The Firing Rate and the rs-fMRI each have an average of five states per individual scan and transition at similar rates, but the average distance between centroids is almost twice as large in the rs-fMRI states compared with the simulated data. Visually the Firing Rate centroids look very similar (Supplementary Figure 4, Kashyap & Keilholz, 2019), suggesting that the diversity of states encountered is still very low. The Kuramoto model produces states with similar distances between centroids as empirical data, but each instantiation seems to have fewer states compared with rs-fMRI. Most runs (66%) result in only a single state, but under certain initial conditions the Kuramoto model can exhibit transitions between two to three different states. The model also seems to dwell in these states longer than in the rs-fMRI and Firing Rate. The transition matrix for the empirical data shows that transitions are more evenly distributed between states than in the simulated data (Figure 6, bottom row). The empirical rs-fMRI data have more transitions between states than in either simulated model. The Kuramoto and the Firing Rate are roughly around the same complexity seen in the transition matrices but far less than seen in the empirical signal. We quantified this by measuring the sparsity fraction by counting the number of transitions and dividing by the total number of possible transitions (Firing Rate has 0.55 transitions, Kuramoto has 0.52, and rs-fMRI has 0.86).

**Figure 7.**
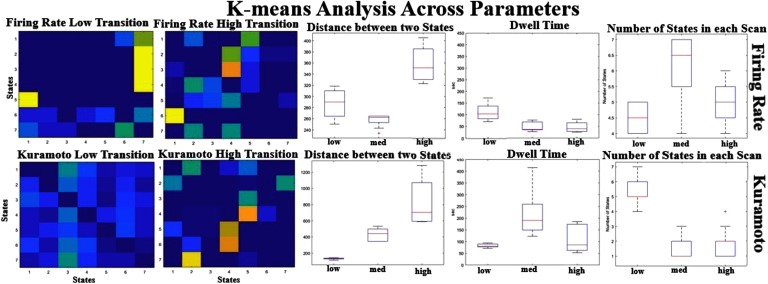
K-means analysis across parameters. The transition matrices for the Firing Rate (top) and the Kuramoto (bottom) are given at different coupling parameters. The distance between cluster centers (middle) seems to be increasing as a function of coupling strength, suggesting that the states are diverging and are having more state-like dynamics for higher coupling strengths. The number of states seems relatively the same even at higher coupling strengths, suggesting that the models are limited to how much variety they can produce.

Varying the coupling strength also affected the state dynamics seen in the models. Figure 7 shows that the distance between the states increases as a function of coupling strength, especially for the Kuramoto model, suggesting that stable dynamical states seem to be moving apart from each other. The other parameters seem to have a nonlinear relationship with the coupling parameters as the dynamics of the system changes. None of the models have the complexity seen in rs-fMRI in terms of number of distinct states and large distances between their centers, but the one that seems the closest to the real signal is the Kuramoto model at medium coupling levels. Even though the BNMs seem to produce some state transition properties, these BNMs clearly have much simpler dynamics as compared with the rs-fMRI even when varying the parameters.

### Recurrence Quantification Analysis (RQA)

**Figure 8.**
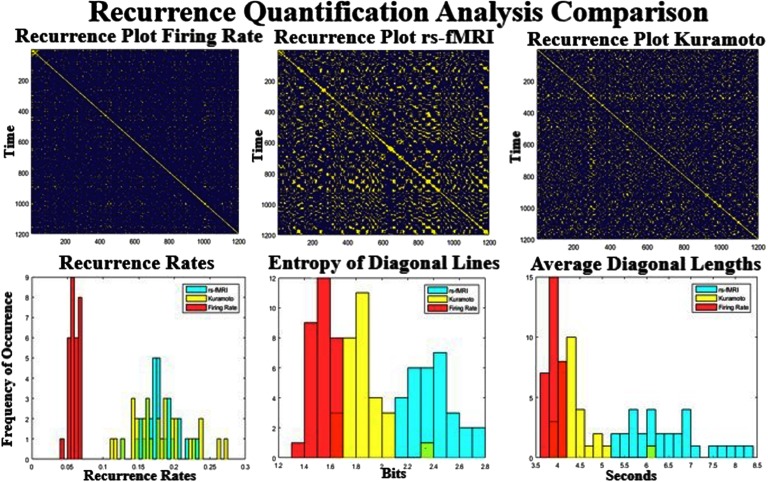
Comparison of the recurrent quantification analysis (RQA) between the simulated and real datasets. The top row shows three scans of recurrent plots thresholded at 0.3 for the three datasets. The bottom row shows the distribution of three different RQA techniques over all scans. The bottom left shows the recurrence rate, which is much higher for the Kuramoto and rs-fMRI model than for the Firing Rate simulation. The recurrence rate is a measure of repeated states seen in the dynamics of the rs-fMRI signal. The middle and the right plots quantify how much similar trajectories occur during the scan. The measured rs-fMRI signal shows much more variance between different trajectories and is of much longer duration.

Figure 8 shows the reccurance plots for the empirical and simulated BOLD signal. These plots are calculated by correlating the pattern of activity at each time point with the pattern from every other time point. Diagonal lines that are parallel to the main diagonal represent repeating transistions that are seen throughout the scan, whereas vertical or horizontal blocks represent dwell periods during the scan. A cursory inspection of the three recurrence plots (Figure 8, top row) shows that the two models have far less repeating structure than seen in rs-fMRI. This relation is quantified by the bottom three plots that show the recurrence rate (left), entropy of diagonal lines (middle), and average length of diagonal lines (right). The reccurence rate seems to be much higher in the Kuramoto and empirical signal than in the Firing Rate model. However, the entropy and length of the lines (related to how different the states are and how long they linger) clearly separate the three datasets (bottom and right). Entropy and line length are highest in the real data and lowest in the Firing Rate simulation, with the Kuramoto simulation residing in between. The low entropy values for the Firing Rate data signify that the model does not have as many repeated trajectories as compared with the other modalitites.

**Figure 9.**
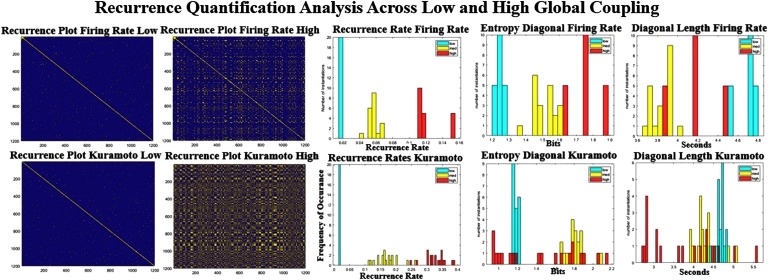
Recurrence quantification analysis across different parameters. Left are the recurrence plots at high and low global coupling for Kuramoto (bottom row) and Firing Rate (top row). The recurrence rate that quantifies the number of events increases monotonically (center column) as a function of coupling strength. The entropy and the lengths of the diagonal lines seem to have a nonlinear relationship. Neither of the right two metrics are as high, as seen in the resting dataset (Figure 8).

Changing the global parameters has some very linear effects, as can be seen in Figure 9. At low coupling parameters the reccurence plot shows almost no structure, and at high coupling more and more structure emerges. The reccurence rate that is most related to the number of events seen in reccurance plots is almost linear (Figure 6B, middle column) for both models. At higher levels of coupling there seems to be a larger spread in the models (entropy rate, Figure 9, right from middle column), as they seem to be more a function of initial condition than at low levels of coupling. The entropy levels and the average diagonal length seem to be much higher in rest than all of the models, suggesting longer, slower repeated trajectories in the real signal. Overall the technique is able to separate the emprical data and the models pretty robustly and shows a clear difference between the more simpler Firing Rate model and the more complex Kuramoto model, and at least one of the measures seems to vary linearly with the parameter selection.

## DISCUSSION

### Average Functional Connectivity and Power Spectra

From previous studies using multiple models and parameterizations, it appears that certain properties of the simulated signal are most reliant upon the underlying structural connectivity rather than the model of activity used at each node (Bullmore & Sporns, 2009; Stam et al., 2016). In our study, these properties should be similar across models (which share identical structural connectivity) and in the real data. Average functional connectivity analysis is one of these properties. The structural connectivity matrices derived from diffusion tensor imaging and the respective functional connectivity estimates derived from resting-state fMRI have a correlation value of 0.45 as measured through our methodology, which is similar to what has been described before (Bullmore & Sporns, 2009). In fact, all three dynamical systems produce signals where functional connectivity is highly correlated with the structural input, leading to suggest that average functional connectivity is closely related to the underlying connectome. There are known differences between SC and FC; for example, an edge in FC between two nodes can be the result of a third process that drives the two structurally disconnected regions. However, most of the edges between the ROIs can be described as a function of how many white matter tracks run between them (Stam et al., 2016). Moreover, the frequency spectrum and the characteristic ${\left(\frac{1}{f}\right)}^{n}$ distribution are similar for both BNMs and the empirical rs-fMRI, suggesting that the spectrum is a property of the underlying structure of the network. The Firing Rate model reproduces the average FC better than the Kuramoto model, which might be due to overfitting since it has fewer parameters to optimize compared with the Kuramoto model. This matches well with previous reported literature where the Firing Rate model has produced FC matrices that have a large correlation (corr. = 0.8) by tweaking the input SC to maximize similarity to the output FC (Senden et al., 2017).

### Coactivation Patterns/Point Process

The coactivation analysis also showed a shared feature across the empirical and simulated data. For all three datasets, coactivation patterns were strongly correlated with average functional connectivity. This is likely because functional connectivity is driven by processes that activate certain subnetworks of the SC together (Smith et al., 2009). This further strengthens the notion that SC and average FC are closely related. The global coupling parameter affects the coactivation rates in a similar way as for average FC, where low levels of coupling result in a very uncoordinated system, and high levels result in global networks that are highly active. Since the model parameters were chosen to fit average FC best, and it is easier to fit the Firing Rate model, it once again reproduces measures that are more faithful to empirical rs-fMRI compared with the Kuramoto model, probably for the same reason that it can better match the average FC of the empirical data. Regardless of the coupling parameters used, the coactivation patterns are similar to the average FC for both models.

### QPPs

The successful detection of quasiperiodic patterns in both BNMs indicates that these network models capture at least some of the dynamical features of the brain’s activity. Unlike the previous sections of spatial metrics where the naïve Firing Rate model performed better than the Kuramoto model, the QPP templates for the two models are indistinguishable. On the other hand, there are substantial differences compared with the real QPP. The real QPP is more complex, with gradual switching at different time lags in different areas. The real QPP template is also longer in length than the ones from the BNMs, as the simulated model starts repeating itself before the end of the template. The length of the spatiotemporal pattern seems to be a function of the coupling parameter, especially in the Kuramoto model, where increased coupling leads to faster repetitions of the patterns. The overall spatial shape of the pattern (i.e., the areas involved in activation and deactivation) are quite similar across models and parameterizations, though none are as complex as the patterns in empirical data. We believe the incorporation of aspects of neural field models and Connectome Harmonics (Atasoy, Donnelly, & Pearson, 2016; Sanz-Leon et al., 2015) into the existing BNMs may result in a more accurate reproduction of the spatial propagation because it would take into account surface propagation was well as network propagation. It could also be possible that the difference in QPP templates results from the unidirectionality of certain white matter connections or other properties that cannot be captured using standard tractography.

### K-Means Analysis

Some dynamic properties appear to arise more from the complex interactions linked to the unique temporal description of activity in each ROI than from the underlying structural connectivity. These properties are likely to be different for each BNM, and from previous literature, the Kuramoto model has outperformed the Firing Rate model (Cabral et al., 2017). The k-means algorithm on the windowed FC matrices revealed a complex network of states in the rs-fMRI data that each demonstrated distinct spatial patterns of connectivity between ROIs along with a complex web of transitions between them. In the Firing Rate model there are a similar number of states compared with rs-fMRI, but the distances between the states are much smaller compared with rs-fMRI, suggesting that it is more appropriate to consider the Firing Rate states as representing a single state artificially divided into multiple components. The state description in the Firing Rate model echoes previous findings because it is known that stable attractor states cannot be produced with only a linear set of differential equations (Cabral et al., 2017). The Kuramoto model at very low levels of coupling has similarly many states that are close together. But at higher values the Kuramoto model, for some initial conditions, can produce transitions between states that are as spatially distinct as the rs-fMRI but limited to fewer states and a simpler transition matrix than the empirical signal. This suggests that BNMs can reproduce at least some of the dynamic states observed in rs-fMRI, although current models do not recapitulate the rich variety observed in empirical data. The Kuramoto model under certain parameters does better than the Firing Rate model and can produce complex state-like behavior.

### Recurrence Analysis

Recurrence analysis quantifies elements in the temporal structure of the data similar to clustering analysis on short FC matrices. Therefore, we predicted it to depend more on the dynamic description of the data rather than the shared spatial connectivity input. The Firing Rate model again exhibited the least complexity, the rs-fMRI data exhibited the most, and the Kuramoto model fell in between the two for the normal parameters. The empirical data have repeated trajectories that occur more often and are longer than either observed in the simulated BNMs. Out of the dynamic analysis metrics that were examined, the RQA metrics separated the three datasets the most effectively, whereas the average functional connectivity analysis exhibited the fewest differences. Moreover, the recurrence rate that quantifies the number of repeated temporal events seems to linearly depend on the coupling parameters for a certain range. This relationship is similar to average FC, which at low levels of coupling shows no structure and at higher levels of coupling shows increased network structure (Supplementary Figure 2, Kashyap & Keilholz, 2019). Average FC and recurrence analysis both use correlation, except that one uses the space across rows that spans the ROIs, whereas the other uses the space of the columns that represent single time points in the BOLD data. Average FC, which examines coordination between ROIs, reveals a static network related to the input SC. Recurrence analysis that examines the time domain reveals properties that seem to be most unique to the formulation of each BNM. Recurrence analysis also is quick to compute and therefore could be a good addition to average FC as a metric for model selection, which is a very computationally intensive process. Together they can ensure that the model has roughly similar network component structure compared with rs-fMRI based on average FC, and similar temporal structure recurrence rates.

### Overall Discussion

The goal of our exploratory study was to find better dynamic metrics to compare empirical rs-fMRI and the brain network models. We have chosen two different BNMs at three different parameterizations to provide an axis of contrast between the simpler Firing Rate model and the more complex Kuramoto model, which has been shown to reproduce more complex dynamic trajectories (Cabral et al., 2017). The dynamic analysis techniques can be ordered in how much they analyze the structure in the spatiotemporal BOLD signal as a function of spatial coordination between regions or repeated temporal trajectories. The ordering in Table 1 is not strict, but loosely goes from techniques that observe spatial patterns to those that observe temporal patterns. These two components seem to correspond to the two main components in the formulation of BNM: the structural network that provides input from connected ROIs and a description of the evolution of the state variables.

**Table 1.** Comparison between models across different analysis techniques.

**Analysis technique**	**Firing rate**	**Kuramoto**	**Metric**
Static FC	Better	Worse	Correlation between average FC of rs-fMRI and models (Figure 2)
Point process	Better	Worse	Correlation between point process matrices of rs-fMRI and models (Figure 3)
QPP	Tied	Tied	Correlation between the shape of extracted spatiotemporal signal between rs-fMRI and models and their rate of occurrence (Figure 4)
K-means	Worse	Better	Number of distinct states and diversity of state transitions (Figure 6)
RQA	Worse	Better	Average number of repeated temporal transitions and the entropy of those transitions (Figure 8)

The Firing Rate model outperforms the Kuramoto model on metrics that are more closely linked with spatial patterns, whereas the Kuramoto performs better on metrics that are linked with the temporal structure observed in rs-fMRI. We believe that the performance on spatial metrics, such as average FC and point process, is due to the Firing Rate model being easier to fit to the rs-fMRI because of its fewer parameters. Moreover, since average FC and the SC are similar, it is probably an easier task to match the FC output that is very closely related to the SC input. The temporal metrics reveal that the Kuramoto model has much richer dynamics than the Firing Rate model and is closer in reproducing features seen in rs-fMRI. Moreover, these differences are more likely due to the differences in the differential equation formulation of the BNM since it defines the network evolution. The BNMs seemed to perform also similarly between the QPP and the point process metric, suggesting that it might be an invariant property of all BNMs.

### Limitations

Our modeling approach makes many simplifying assumptions that do not capture the true complexity of the brain. In the construction of the structural connectome, we assumed that all connections were bidirectional. This is a limitation of using tractography to build the structural network, since tractography cannot distinguish unidirectional connections. Moreover, estimates of fiber density for connections between regions that have very sharp angles or between regions that are spatially far apart are far lower than the true connectivity between these regions (Bullmore & Sporns, 2009). In our generative models we also assumed a homogeneity in the response of ROIs, in both their neural description, as well as their transformation using the hemodynamic Balloon-Windkessel model. Moreover, we did not simulate subcortical structures that are known to play a crucial role in the operation of the central nervous system. All these factors might change the association between dynamic metrics and the simulated BNM signal.

We also examined only a single parameterization for only two BNMs. There are a variety of BNMs, some of which are likely to exhibit more complex dynamics than either the Kuramoto or the Firing Rate model (Sanz-Leon et al., 2015). Even different parameterizations of a single model can give rise to vastly different behavior (Hansen et al., 2015). We chose to focus on the Kuramoto and Firing Rate models because of their relative simplicity, their thorough characterization, and the expectation that they would have dissimilar dynamic properties.

There are also numerous dynamic analysis methods available for rs-fMRI (Keilholz et al., 2017). We chose to focus on a few of the most common ones, but future work should certainly examine the use of other types of analysis to produce even more sensitive metrics. Moreover, our study does not look at methods to test these metrics and use the established correlation as distance function. We have also not explored the entire space of parameterization, so it is possible that these models can produce more realistic signals; however, based on previous results establishing these as close to optimum, the results are probably a realistic representation of their capabilities.

### Conclusion

We believe that either of the two more-temporal metrics, namely RQA or k-means, would be the most appropriate in evaluating BNMs in the future. The k-means approach is a stronger criterion to evaluate on, because the cluster centers as well as the state transitions between the model and the empirical signal would have to match in order to reproduce resting-state dynamics. However, the RQA approach is less computationally intensive and can used to quickly check the diversity in the temporal structure of the simulation and to assist the selection of parameters in the model. The QPP algorithm would be useful in evaluating properties of the network structure, as it seems to be common between the BNM. It probably would be more useful to evaluate models that incorporate more biophysical plausibility such as neural field models and connectome harmonics. The average FC and the point process do not reveal processes that are much more complex than the SC input, but they useful in the way they are currently used to sweep the parameters and bias the system.

From the dynamic analysis perspective, the most distinguishable metric in rs-fMRI seems to be predicting the temporal structure of the signal. We already know how the different ROIs are connected as a network, but it is still a mystery how the signal evolves. BNM provides a mathematical framework for exactly how this signal might evolve, so it is appropriate to evaluate them by characterizing their temporal dynamics.

## METHODS

### Structural Connectome

Using Human Connectome Project’s diffusion-weighted images (spin echo TR 5520 ms, TE 89.5 ms, flip angle 78, voxel 1.25 mm) from five random subjects (Van Essen et al., 2013), we generated one average structural connectome. Tractography was performed using the freely available software MRtrix with maximum fiber length set to 250 mm (Tournier, Calamante, & Connelly, 2012) and parcellated using the Desikan-Killiany atlas (Desikan et al., 2006). For each subject, their respective T1w images (TR 2,400 ms, TE 2.14, voxel size 0.7 mm) were aligned to the standard space; then the using the warping matrix we transformed the diffusion-weighted images. Probabilistic tractography then was run between each ROI and then pruned to generate 10 million fibers. To generate the estimates for the length and weight matrices from the tractography, we used the same methodology as Hagmann et al. (2008). The length between two ROIs was defined as the average fiber length of all fibers that went between them, and the weight was the number of fibers going between two ROIs normalized by the surface area of the receiving ROI. The atlas provides 84 cortical and subcortical ROIs, but we selected the same 66 cortical regions as in Cabral et al. (2011) for comparison to previous work. The resulting matrices are shown in Supplementary Figure 5 (Kashyap & Keilholz, 2019). There are a few important differences between our tractography, the one from Hagmann et al. (2008), and the ideal tractography. Our current tractography, due to longer fiber lengths, has more interhemispheric connections than the one presented in Hagmann et al. (2008). However, tractography is less sensitive to longer connections (Fornito, Zalesky, & Breakspear, 2013) and therefore the between-hemispheric connections were scaled by a factor of 4 to offset the known issue. Tractography is also less sensitive to fibers with sharp angles than to fibers with more straight angles, so for example it results in less connections between the two primary visual areas (ROIs 27–29) that have the sharpest bend in the corpus callosum (Fornito et al., 2013). The final weight matrix was normalized using the matrix norm function to be unit norm. The length matrix was divided by the mean conduction velocity 5.45 m/s to get the delay matrix. This set the mean delay to 11 ms in accordance with Cabral et al. (2011).

#### BNMs.

Brain network models describe the BOLD signal as the coupling of *n* distinct neural populations corresponding to different cortical regions. Each population is connected via a weight matrix obtained from structural connectivity that describes the strength of the connection between nodes. In general each of these *n* areas are modeled by a differential equation for each node: $\frac{dn(t)}{dt}=f(N(t),W,L)$, where *N*(*t*) is the time series of all the nodes/ROIs, *W* is the weight matrix, *L* is the length matrix, and for given random initial conditions for *n*_0_, the time series *n*(*t*) can be solved for by using the Euler integration method (Sanz-Leon et al., 2015). The time series *n*(*t*) is the state variable and is representative of a measurable property of the neural mass such as firing rate. Some variants use more than one variable to represent the state of the neural mass, but in this paper we consider two models that only use one state variable, namely the Firing Rate and the Kuramoto models. Table 2 shows the mathematical description as well as the values of the parameters used in the simulations.

**Table 2.** Parameters

**Parameter name**	**Differential equations that were simulated via Euler method with a time step of 0.1 ms**	**State variables**	**C_np_ – structural network weight**	**T_np_ – structural network delays**	***σ*_n_ – *SD* of Gaussian white noise**	***ω*_*n*_ – oscillator frequency**	***c*_1_ – first eigenvalue of weight matrix**	***τ*_0_ – relaxation constant**
Firing Rate	*τ*_0_$\frac{dr_{n}}{dt}$ = −*r*_*n*_(*t*) + $\frac{k}{c_{1}}\overset{N}{\underset{p=1}{\sum }}$ *C*_*np*_*r*_*p*_(*t* − *τ*_*np*_) + *σn*(*t*)	r(t) – mean firing rate	K = 0.9 (scale of Cnp)	11 ms set for mean	2 rad/s	N/A	calculated	20 ms
Kuramoto	$\frac{d\theta_{n}}{dt}$ = *ω*_*n*_ + *k* $\overset{N}{\underset{p=1}{\sum }}$ *c*_*np*_ sin(*θ*_*p*_(*t* − *τ*_*np*_) − *θ*_*n*_(*t*)) + *σn*(*t*)	*θ*(t) – oscillator phase	K = 13 (scale of Cnp)	11 ms set for mean	2 rad/s	Randomly initialized as N ∼ (60 Hz, 2 Hz)	N/A	N/A

The Kuramoto model is derived from an assumption that each neural population is in a closed periodic trajectory in phase space that represents its computational processing (Cabral et al., 2011). It has been shown that it can then be modeled by a phasic oscillator that can be described by a single parameter, theta, that represents its location within a 2pi cycle. Inputs into these phasic oscillators perturb its trajectory, but it stays within its limit cycle. Each of these oscillators couples via the network and is driven to the same angle and thus synchronizes the oscillators as a function of the difference between the angles of neighboring oscillators.

The Firing Rate model assumes that the mean firing rate of the neural populations is distributed in a Gaussian manner. This assertion is in accordance with the central limit theorem, which states that the sum of uncorrelated random processes converges to a Gaussian probability distribution, even if the individual processes are highly non-Gaussian. Inputs into this neural mass shift the mean firing rate to a higher firing rate. The mass shifted from its equilibrium tries to relax at the rate proportional to its own firing rate, keeping the system stable via negative feedback.

For each model, the differential equations were numerically integrated with a time step function of 0.1 ms for a duration of 15 min to match the length of an HCP rs-fMRI scan. The first 20 s are thrown away to avoid transient effects from initial conditions. The choices for the values for all the parameters given in Table 2 follow previous work by Cabral et al. (2012) and Cabral et al. (2011), except that the values for k are slightly different than the ones in the paper to account for differences in the structural connectivity matrix. The values were slightly smaller for the Kuramoto (13 instead of 18) because there were more numerous connections in the newer tractography. The low coupling models were simulated for the Firing Rate at k = 0.3. The Kuramoto model had a low coupling of 3. The high coupling models were simulated at k = 60 for Kuramoto and k = 0.999 for Firing Rate. We simulated 30 individual runs at parameterization values from previous studies, and 20 runs each at high and low global coupling levels. Simulations of functional connectivity and the intermediate steps with the original Hagmann matrices and the comparisons with Cabral et al. (2011) are given in Supplementary Figure 1 (Kashyap & Keilholz, 2019).

#### Converting to BOLD.

In order to compare the neural simulated data with the hemodynamic response measured from fMRI, we have to convert the high-frequency activity down to the low-frequency hemodynamic response. This is performed with the Balloon-Windkessel model, which is a quadruple differential equation model that in a neuronal input and calculates the blood flow and blood volume and uses that to estimate the fraction of the oxygenated blood to the deoxygenated blood (Friston, Harrison, & Penny, 2003; Stephan, Weiskopf, Drysdale, Robinson, & Friston, 2007). Supplementary Figure 6 (Kashyap & Keilholz, 2019) shows the impulse response of the Balloon-Windkessel model, which looks roughly like the canonical hemodynamic response function. We used the same constants for our Balloon model as those given in Friston et al. (2003). After passing the output of the BNMs through the Balloon-Windkessel model, it was then downsampled to the same sampling rate as the rs-fMRI data (0.72 s).

#### Pre-processing rs-fMRI.

For the rs-fMRI data we used 30 individual HCP scans (gradient echo EPI, TR 720 ms, TE 33.1 ms, flip angle 52, voxel 2 mm) that are each roughly 15 min long. The data came from the minimally processed pipeline and then were ICA denoised using the 300 ICA vectors that HCP provides. We then applied the same Desikan-Killiany atlas as used in the tractography onto the data and obtained the mean time series for each ROI. From then on, the same processing pipeline was applied for the simulated data and the real data, in order to keep the processing as similar as possible. These steps in order were z-scoring each time series, then band passing filtering the signal from 0.01 to 0.25 Hz, then global signal regression using a linear regression model, and then applying a final z-score step. These steps were selected in accordance with Cabral et al. (2011).

### Dynamic Analysis Techniques

To compare the dynamics of the rs-fMRI signal and the BNMs, we selected analysis techniques that are commonly used and characterized the signal at different spatial and temporal scales. Table 3 shows a quick comparison of the different techniques that were applied.

**Table 3.** Dynamic analysis techniques

**Analysis technique**	**Description**	**State variable**	**Spatial scale**	**Temporal scale**
Point process/neural avalanche	FC is driven by discrete events in rs-fMRI when different ROIs coactivate together	Level of activity of a single ROI	Very similar to average FC and the SC input	Coordination is seen in the shortest scales (∼ 1 s)
Quasiperiodic pattern	A specific spatiotemporal pattern observed in rs-fMRI that repeats itself over the length of the scan	Phase during the spatiotemporal pattern template	Specific nodes go through a sequence of activation and inactivation	Pattern (20 s) Occur (2/3 per min)
Recurrent quantification analysis	A sequence of similar ROI activity and/or trajectories that appear to repeat over time	Level of activity across the whole network at a single point in time	Repeating firing patterns over time	Medium (∼ 10–20 s)
K-means clustering of FC matrices	Multiple stable FC states that transition between each other	Windowed FC matrices at ∼ 45 s	Local networks embedded in the larger SC that coordinate processing for a stable period of time	Longest (45 s–1 min)

The point process assumes that activity in an area triggers neural avalanches in regions that are involved in the information processing (Tagliazucchi et al., 2012). The signal is only interpretable at either its high levels of activation or very low levels of activation when it is coordinating information transfer with other elements in the network. Later models explicitly write out the mathematical formulation using impulse response and solve for a sparse representation of these coactivation patterns, which are thought to be unique computational trajectories across the brain (Karahanoğlu & Van De Ville, 2015; Liu & Duyn, 2013). But in this analysis, we use Tagliazucchi’s methodology by quantifying when different ROIs cross the same threshold over time. We implemented this approach by recording when the activity at a certain ROI crosses a certain threshold and then counting how many other ROIs cross the same threshold within three time steps (0.72 s) of the original crossing. We normalize the co-occurrence rates to get a fraction by dividing by the total number of crossings at each ROI. We applied this analysis with two different thresholds, one at the mean of the signal and one at 1 standard deviation away, which for our normalized signals were at 0 and 1, respectively. Prior work that has shown that average functional connectivity is primarily driven by coactivation events (Tagliazucchi et al., 2012).

A second approach examines the quasiperiodic patterns of BOLD signal propagation over the course of the scan. The QPP algorithm identifies the most prominent repeating spatiotemporal pattern in the signal (Majeed et al., 2011; Majeed et al., 2009). In brief, the algorithm chooses a random chunk of the rs-fMRI data (20 s of data) and correlates it with the entire scan (Majeed et al., 2011). Time points with high correlation to the random chunk indicate repeated occurrences and are averaged together to form the new template. This process is iterated until the template converges. Since we use a random seed point as the original template, repeated runs of the algorithm produce QPPs with different phases. Therefore, in order to compare the patterns from the rs-fMRI and the simulated models, the QPP was circularly shifted to the point where maximum correlation occurred.

Sliding window correlation followed by k-means clustering was applied to examine the brain states and transitions in each set of data (Allen et al., 2014). Using a sliding window length of 60 × 0.72 s, the Pearson correlation was calculated pairwise for all ROIs. The window was then advanced by one time point and the process was repeated until the window reached the end of the scan. This value is around the range used in previous work (Allen et al., 2014). Correlation values were Fisher-transformed to better approximate a normal distribution and the k-means algorithm was applied to cluster the data into seven groups using Manhattan distance based on previous studies (Allen et al., 2014). Clustering was repeated 30 times, and the best resulting clustering was chosen based on minimizing the total distance from the cluster centroids and the feature vectors in order to mitigate the effects of randomly choosing the centroid locations.

Recurrent analysis was performed by calculating correlation of the spatial pattern of activity pairwise across all time points. We then thresholded the values at 0.3, based on literature search, and created recurrent plots (Bassett, Nelson, Mueller, Camchong, & Lim, 2012; Cabral et al., 2014). These metrics were calculated using freely available MATLAB toolbox (Ouyang, Li, Dang, & Richards, 2008), and their distribution for each type of data was plotted. Recurrence rate, entropy rate, and average diagonal length were measured. The recurrence rate is the rate that similar states occur throughout the scan, as seen in Equation 1. Entropy rate quantifies the difference between repeated states, as seen in Equation 2. The average diagonal length, Equation 3, measures how long these trajectories occur. Collectively they give us an insight into how often similar states occur, how different they are from each other, and how long each of these spatiotemporal trajectories persists.

$$RR=\frac{1}{N^{2}}\sum_{i,j=1}^{N}R(i,j),\;\text{where}\;R\;\text{is}\;\text{the}\;\text{recurrent}\;\text{plot}.$$ (1)

$$L=\frac{\overset{N}{\underset{l=lmin}{\sum }}lP(l)}{\overset{N}{\underset{l=lmin}{\sum }}P(l)},\;\text{where}\;P(I)\;\text{is}\;\text{the}\;\text{frequency}\;\text{of}\;\text{diagonal}\;\text{length}\;I.$$ (2)

$$ENTROPY=-\sum_{l=lmin}^{N}P(l)\ln(P(l)),\;\text{where}\;P(l)\;\text{is}\;\text{the}\;\text{frequency}\;\text{of}\;\text{diagonal}\;\text{length}\;l.$$ (3)

## ACKNOWLEDGMENTS

We would like to thank the help of Dr. Constantine Dovrolis, who provided invaluable help and insight with developing and debugging our initial brain network models. We would also like to thank members of the Keilholz Lab, specifically Behnaz Yousefi and Anzar Abbas, who provided their expertise in the quasiperiodic patterns algorithm, and Dr. Jacob Billings, who helped set up all the servers and the data from the Human Connectome Project that were used in this manuscript.

## AUTHOR CONTRIBUTIONS

Amrit Kashyap: Conceptualization; Data curation; Formal analysis; Funding acquisition; Investigation; Methodology; Project administration; Resources; Software; Validation; Visualization; Writing – original draft; Writing – review & editing. Shella Keilholz: Conceptualization; Data curation; Formal analysis; Funding acquisition; Investigation; Methodology; Project administration; Resources; Supervision; Validation; Visualization; Writing – original draft; Writing – review & editing.

## FUNDING INFORMATION

Shella Keilholz, Georgia Institute of Technology (http://dx.doi.org/10.13039/100006778), Award ID: GT FIRE. Amrit Kashyap, National Institutes of Health (http://dx.doi.org/10.13039/100000002), Award ID: TL1 TR000456-11. Shella Keilholz, National Institutes of Health (http://dx.doi.org/10.13039/100000002), Award ID: R01MH111416.

## TECHNICAL TERMS

Resting-state fMRI (rs-fMRI): Brain activity measured with fMRI while participants lie still with their eyes open and allow their minds to freely wander. The term “resting state” is used to highlight the contrast with task-based fMRI, where participants are actively engaged in a known, well-defined task.
Functional connectivity: The definition of a network based on coordinated activity between different regions. There are two types of functional connectivity: average or static functional connectivity, which is estimated across long scans, and dynamic or transient functional connectivity,which is calculated over short time segments.
Regions of interest: The cortical sheet is parcellated into many different nonoverlapping regions of interest. They cover the whole brain, and each of these regions corresponds to areas that are thought to functionally process similar types of information, that is, visual area 1 (V1) and motor area 1 (M1).
Structural connectivity: The structural network defined by the white matter tracks between different cortical areas. This is usually constructed from diffusion-weighted MRI and is averaged across many individuals.
Brain network model: A network-based model that is used to simulate spontaneous brain activity. It is composed of two main components, the structural network and the activity of each node based on its neighbors.
Dynamic analysis techniques: Different computational algorithms that are applied to resting-state data in order to understand transient features that occur during the scan. They primarily are used to analyze network-based coordination that changes on a moment-to-moment basis.
