# Supplementary material for: Dynamic properties of simulated brain network models and empirical resting-state data
Source: Netw Neurosci. 2019 Feb 1;3(2):405–26. doi: 10.1162/netn_a_00070 (PMC6370489; doi:10.1162/netn_a_00070)
Supplement: Supplementary file 1 [file netn-03-405-s001.pdf]

Kashyap, A. & Keilholz, S. (2019). Supporting Information for "Dynamic Properties of Simulated Brain Network Models and Empirical Resting State Data." *Network Neuroscience*, *3*(2), 405–426.
https://doi.org/10.1162/netn_a_00070

Supplementary Figures:

Table of Regions of Interest (Reproduced from Cabral et al., 2011):

Abbreviation	**ROI/ Brain Region**
ENT	***Entorhinal cortex***
PARH	***Parahippocampal cortex***
TP	***Temporal pole***
FP	***Frontal pole***
FUS	***Fusiform gyrus***
TT	***Transverse temporal cortex***
LOCC	***Lateral occipital cortex***
SP	***Superior-Parietal cortex***
IT	***Inferior temporal cortex***
IP	***Inferior-Parietal cortex***
SMAR	***Supramarginal gyrus***
BTST	***Bank of the superior temporal sulcus***
MT	***Middle temporal cortex***
ST	***Superior temporal cortex***
PSTC	***Postcentral gyrus***
PREC	***Precentral gyrus***
CMF	***Caudal middle frontal cortex***
POPE	***Pars opercularis***
PTRI	***Pars triangularis***
RMF	***Rostral middle frontal cortex***
PORB	***Pars orbitalis***
LOF	***Lateral orbitofrontal cortex***
CAC	***Caudal anterior cingulate cortex***
RAC	***Rostral anterior cingulate cortex***
SF	***Superior frontal cortex***
MOF	***Medial orbitofrontal cortex***
LING	***Lingual gyrus***
PCAL	***Pericalcarine cortex***
CUN	***Cuneus***
PARC	***Paracentral lobule***
ISTC	***Isthmus of the cingulate cortex***
PCUN	***Precuneus***
PC	***Posterior cingulate cortex***

Comparison of our methodology with the original BNM Kuramoto Model

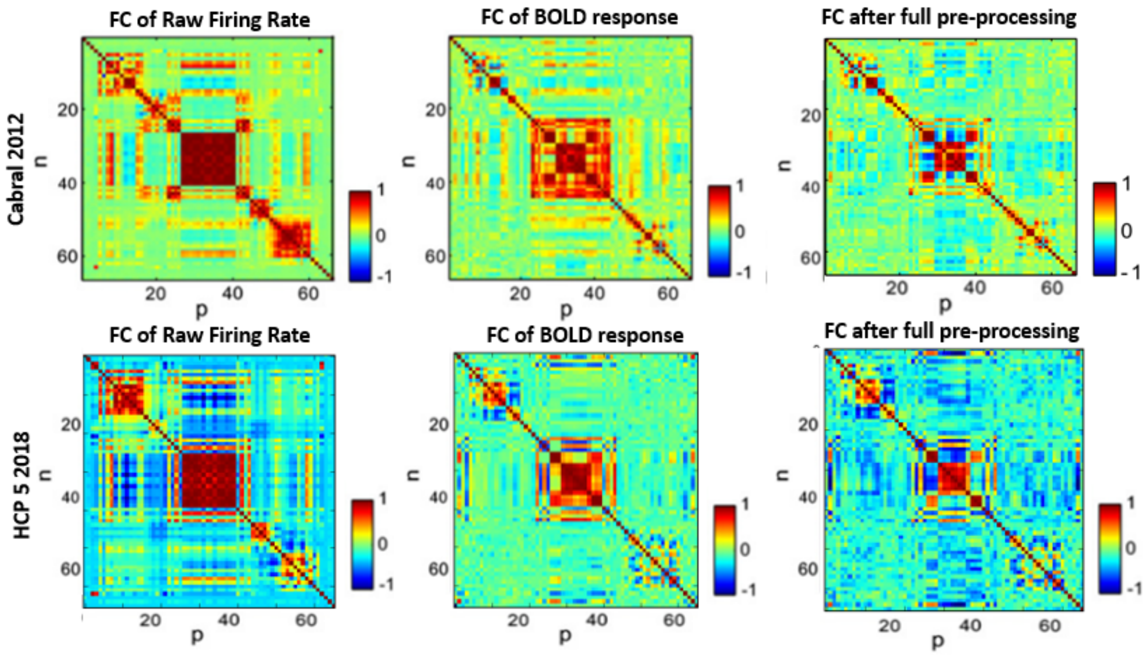

Supplementary Fig 1 – A comparison between all the intermediate states of generating the simulated BOLD signal for the Kuramoto fast oscillator model. The top row is a screenshot from Cabral 2012 paper and the bottom is our reproduction of it using our own methodology. The left most panel represents the functional connectivity matrix calculated from the raw output of the Kuramoto. The middle panel represents the functional connectivity from the output of the Balloon Windkessel model. The rightmost panel represents the functional connectivity using the post processing steps cited in the paper. These models were generated using the 2008 Hagmann structural connectivity which was subsequently replaced by our tractography.

Average FC and Point Process across Parameters

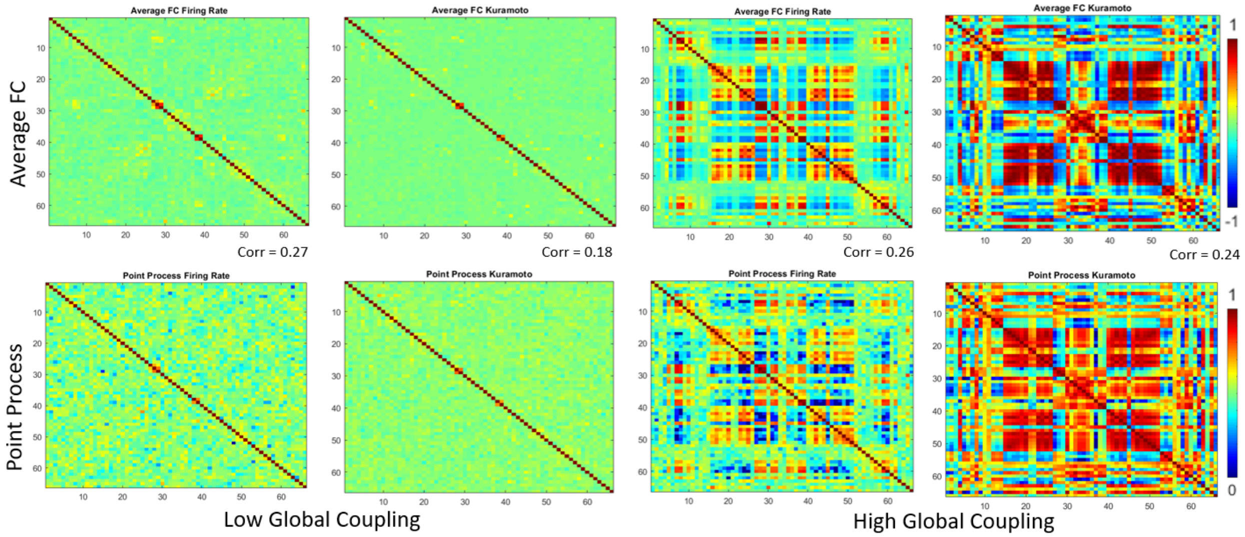

Supplementary Fig 2 – Changes in average functional connectivity and point process for different global coupling parameters. The coupling changes the dynamics from purely noise driven to purely network driven. Average FC is used currently as a metric to find the correct parameter to fit the models to the data. The corr values reflect the correlation to the resting state functional connectivity in Fig 1.

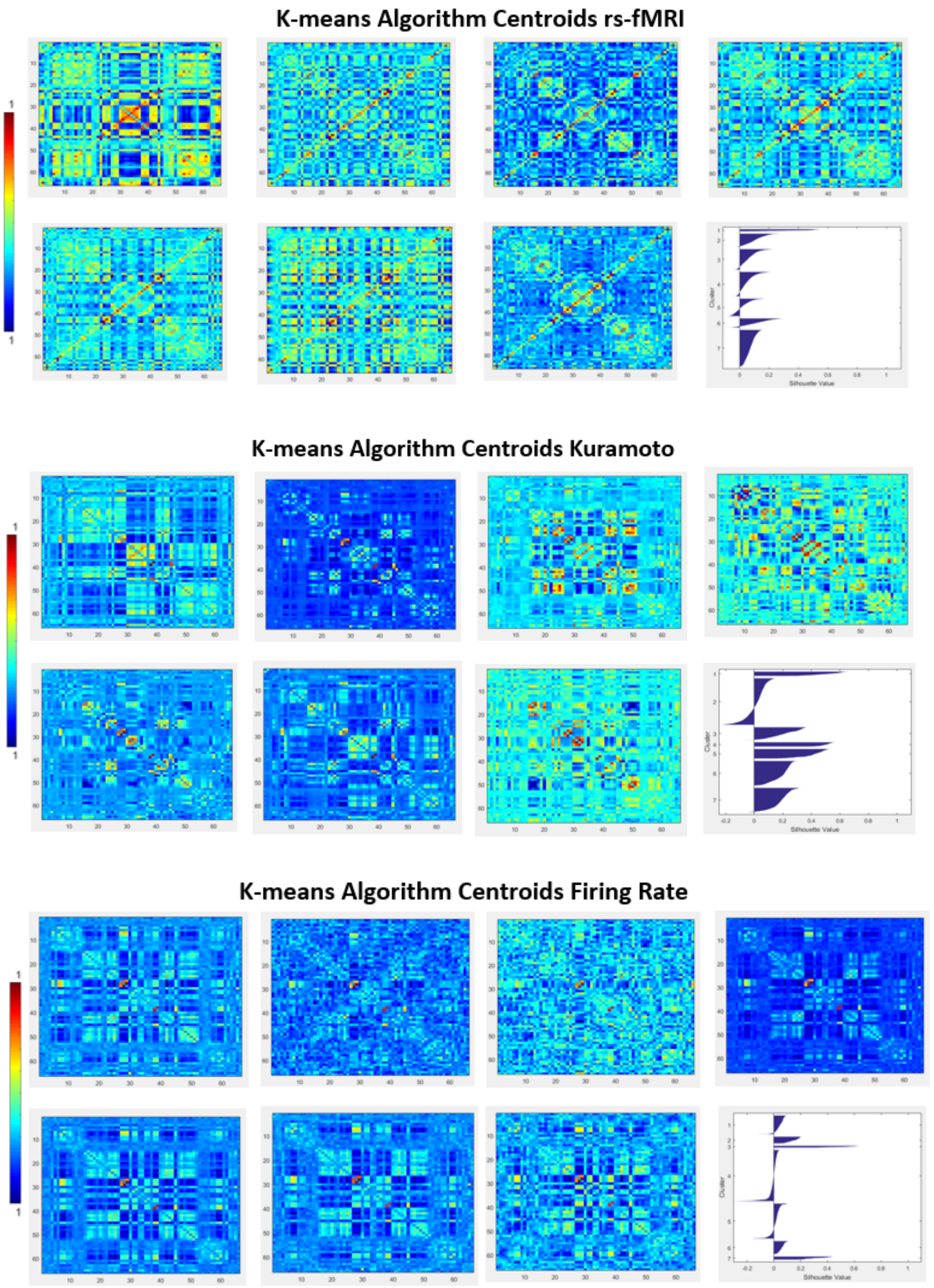

Supplementary Figure 3 - A comparison of all the cluster centroids identified by the K-means algorithm from each of the respective data sets. The spatial states don't vary that much in the two simulated models as much as in the real data. The silhouette (bottom right of each data type) is a measure of how similar the values in each cluster are too each other.

K-means Analysis with different number of Clusters

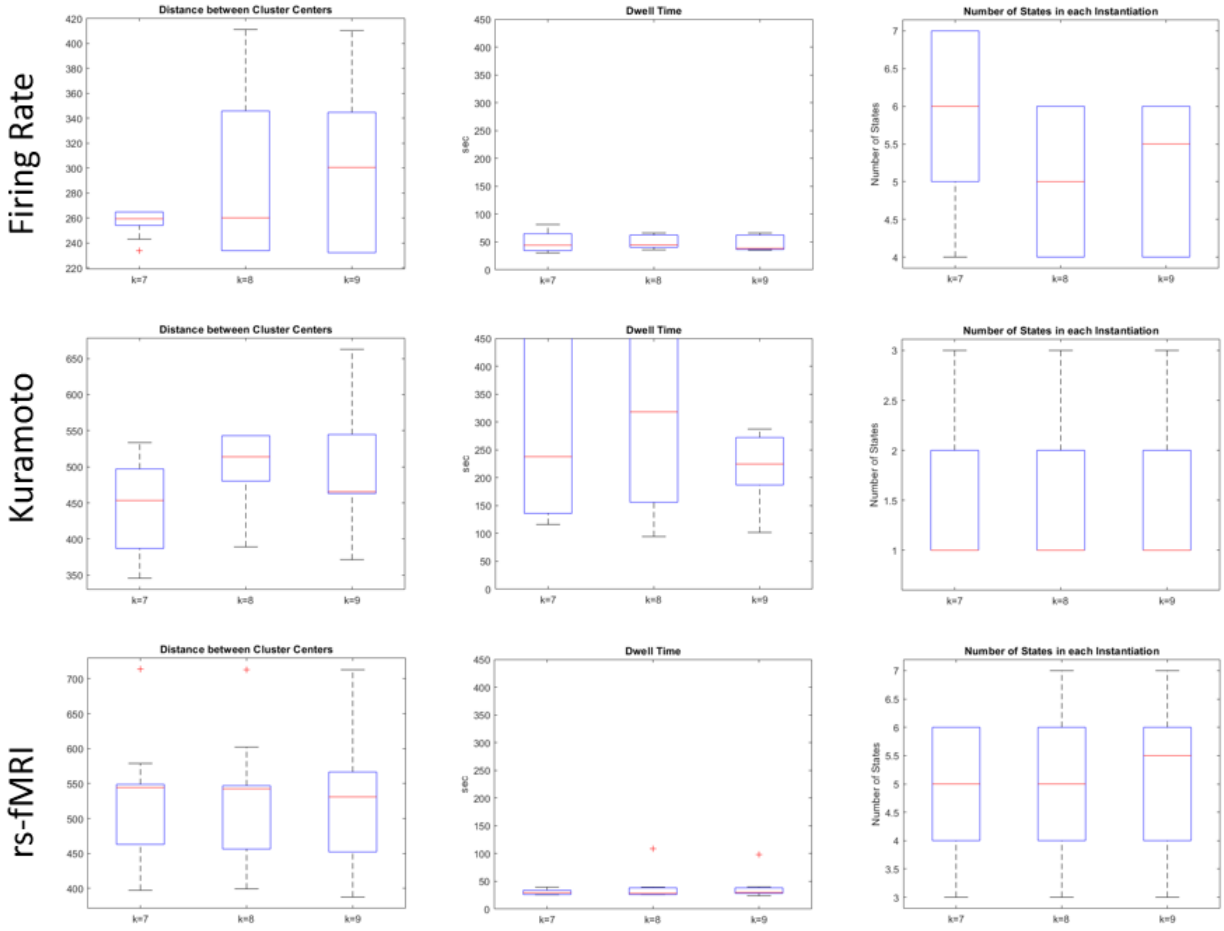

Supplementary Figure 4 – The Hemodynamic Response Model. The equations are a reprint of (Stephan et al., 2007) used in Dynamic Causal Modeling to relate neuronal signal to the output. The model implemented is a set of five differential equations as shown in the diagram with the given variable values. An impulse response is plotted top right and is similar to the canonical Hemodynamic Response function in both its shape and duration.

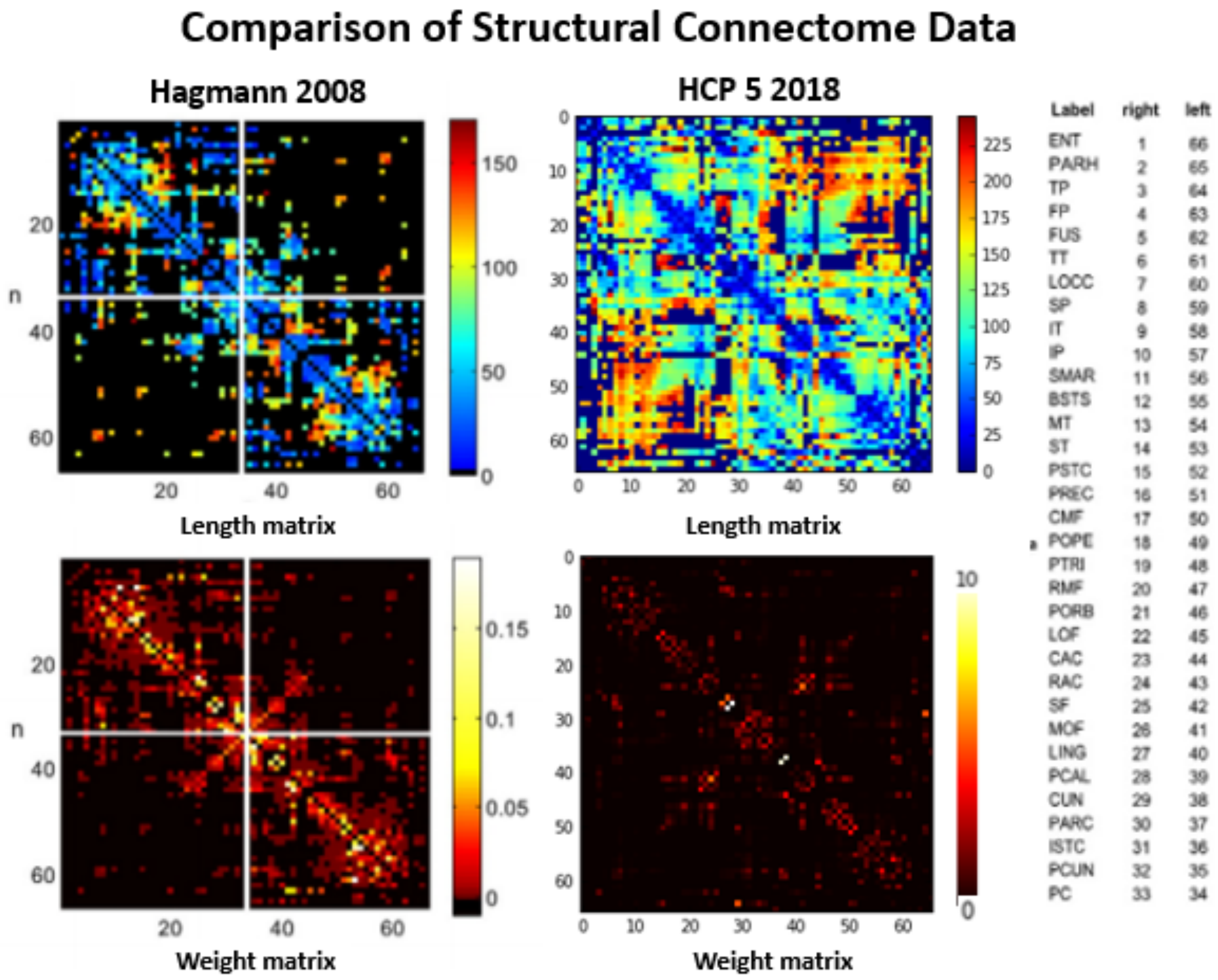

Supplementary Figure 5 – Comparison of our tractography with the tractography that is commonly used by Hagmann et al., 2008. Our tractography has set the max tract length to 250 mm, which allows us to image the longer tracts that are between hemispheres. Top row matrix of the mean length of fiber between two ROI regions measured in mm. Bottom row – number of fibers between two ROI regions divided by the surface area of the receiving ROI (row -> col) and then normalized to one. Left – Hagmans matrices, and right the corresponding matrices from our tractography.

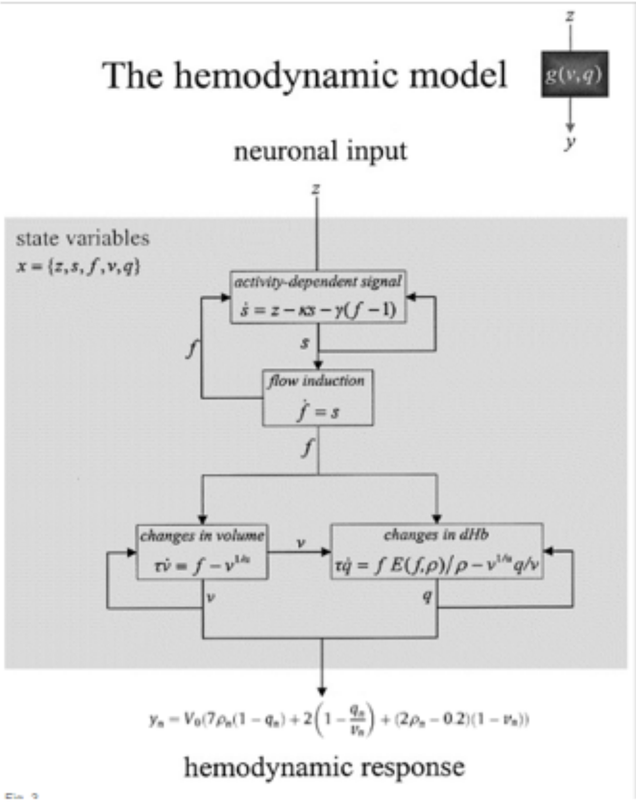

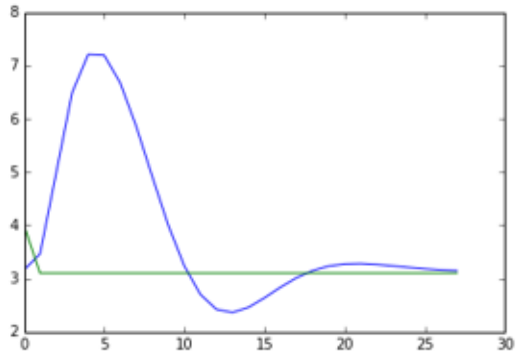

Parameter	*value*
κ	0.65
ρ	0.34
α	0.33
τ	0.98
γ	0.41
V_0	0.02

Supplementary Figure 6 – The Hemodynamic Response Model. The equations are a reprint of (Stephan et al., 2007) used in Dynamic Causal Modeling to relate neuronal signal to the output. The model implemented is a set of five differential equations as shown in the diagram with the given variable values. An impulse response is plotted top right and is similar to the canonical Hemodynamic Response function in both its shape and duration.
